# Human Microglia–Like Cells Differentiated from Monocytes with GM-CSF and IL-34 Show Phagocytosis of α-Synuclein Aggregates and C/EBPβ-Dependent Proinflammatory Activation

**DOI:** 10.1007/s12035-024-04289-z

**Published:** 2024-06-20

**Authors:** Andrea Llaves-López, Elia Micoli, Carla Belmonte-Mateos, Gerard Aguilar, Clara Alba, Anais Marsal, Marta Pulido-Salgado, Neus Rabaneda-Lombarte, Carme Solà, Joan Serratosa, Jose M. Vidal-Taboada, Josep Saura

**Affiliations:** 1 Biochemistry and Molecular Biology Unit, Department of Biomedical Sciences, School of Medicine, University of Barcelona, IDIBAPS, Casanova 143, 08036 Barcelona, Catalonia Spain; 2 Department of Neuroscience and Experimental Therapeutics, IIBB, CSIC, IDIBAPS, Barcelona, Catalonia Spain; 3 Peripheral Nervous System, Neuroscience Department, VHIR, Vall d’Hebron Research Institute, Barcelona, Catalonia Spain; 4 Institute of Neurosciences, University of Barcelona, Barcelona, Catalonia Spain

**Keywords:** Microglia, Cell culture, Monocyte-derived, Neuroinflammation, In vitro model, C/EBPbeta

## Abstract

Microglia, the main resident immune cells in the central nervous system, are implicated in the pathogenesis of various neurological disorders. Much of our knowledge on microglial biology was obtained using rodent microglial cultures. To understand the role of microglia in human disease, reliable in vitro models of human microglia are necessary. Monocyte-derived microglia-like cells (MDMi) are a promising approach. This study aimed to characterize MDMi cells generated from adult human monocytes using granulocyte–macrophage colony-stimulating factor and interleukin-34. To this end, 49 independent cultures of MDMI were prepared, and various methodological and functional studies were performed. We show that with this protocol, adult human monocytes develop into microglia-like cells, a coating is unnecessary, and high cell density seeding is preferable. When compared to monocytes, MDMi upregulate the expression of many, but not all, microglial markers, indicating that, although these cells display a microglia-like phenotype, they cannot be considered bona fide human microglia. At the functional level, MDMi phagocytose α-synuclein aggregates and responds to lipopolysaccharide (LPS) by nuclear translocation of the transcription factor nuclear factor-kappaB (NFkappaB) and the upregulation of proinflammatory genes. Finally, a long-lasting silencing of the transcription factor CCAAT/enhancer protein β (C/EBPβ) was achieved by small interfering RNA, resulting in the subsequent downregulation of proinflammatory genes. This supports the hypothesis that C/EBPβ plays a key role in proinflammatory gene program activation in human microglia. Altogether, this study sheds new light on the properties of MDMi cells and supports these cells as a promising in vitro model for studying adult human microglia–like cells.

**Supplementary Information:**

The online version contains supplementary material available at 10.1007/s12035-024-04289-z.

## Introduction

Microglial cells are a CNS-specific immune cell type that plays various important roles in physiological conditions but acquire their most relevant functions in response to stress [1]. Microglia are claimed to be involved in many neurological and psychiatric disorders such as Alzheimer’s disease and schizophrenia [2, 3]. In most neuropathological conditions, they show a pro-inflammatory and oxidative phenotype which is thought to contribute to neuronal damage [4]. The shift from homeostatic to proinflammatory microglia entails massive changes in gene expression [5] and is orchestrated by a small subset of transcription factors [6], including NFkappaB and C/EBPβ. Data from our group show that C/EBPβ regulates this response in mouse microglia [5, 7]. However, whether this transcription factor plays also this role in human microglia has not yet been determined. Another important feature of microglia in relation to disease is its phagocytic capacity. Defective phagocytosis by microglia of misfolded protein aggregates, such as amyloid-beta (Aβ) or α-synuclein deposits, may lead to the accumulation and propagation of these misfolded proteins, leading to pathogenesis [8, 9].

In recent years, novel methods have enabled us to investigate the role of microglia using in vivo models [10]. However, most of our knowledge about their properties and function has been obtained with cultured microglial cells. The most popular methods to study microglial function are primary microglial cultures isolated from mixed glial cultures by a variety of strategies including shaking [11], mild-trypsinization [12], or magnetic cell sorting [13]. It is important to note that, although rodent and human microglia share many properties, significant differences exist [14, 15]. For instance, expression of nitric oxide synthase-2 (NOS2) is much higher in rodents than in human microglia [16, 17], which is a relevant difference since NOS2 is a key element in neuroinflammation-induced neurotoxicity in vitro [18–20]. In fact, there is a greater overlap in transcriptional profile between stimulated primary human microglia and human monocytes than with mouse microglia [21]. In order to understand the role of microglia in human disease it is therefore essential to be able to work with microglial cells of human origin.

There is no golden standard protocol to obtain cultures of human microglial cells, but several approaches exist. Primary cultures are generally considered the best option since they are the closest to the in vivo situation. Primary human microglial cultures can be prepared from fetal [22, 23] or adult tissue, either resected in neurosurgery [23, 24] or obtained post-mortem after rapid autopsy [22, 25]. However, these methods often yield limited cell numbers, have low microglial enrichment in the final culture, and present ethical and practical challenges. Consequently, alternative protocols for obtaining human microglial cultures have been explored for decades. One promising approach involves the differentiation of readily available adult human cells into cells with a microglial phenotype, often called microglia-like cells. On the one hand, several protocols have been published to differentiate human-induced pluripotent stem cells (iPSC) into microglia-like cells. With this approach, iPSCs are first derived into hematopoietic progenitors by combinations of growth factors. Subsequently, these progenitors are differentiated into microglia-like cells through co-culture with other cells, conditioned media, and/or cytokines[26]. While these protocols are promising, they are laborious and long. On the other hand, several groups have described protocols to differentiate adult human monocytes into microglia-like cells. To favor a microglial phenotype, these protocols use co-culture with astrocytes [27], astrocyte-conditioned medium [28], cytokines, and growth factors with microglial pro-survival and differentiating properties such as M-CSF, GM-CSF, IL-34, NGFβ, and CCL2 [29, 30], or a combination of these strategies [31, 32]. Since monocytes and microglia share the same lineage, these protocols are simpler and shorter than those starting from human stem cells, and they have also the advantage of the relatively easy availability of adult human monocytes from peripheral blood. They are a particularly suitable model for comparing microglia-like cells between controls and patients [31, 33] or for studying microglia-like cells with disease-relevant genetic variants [29, 30]. An excellent systematic review has recently compared the different published protocols to prepare human monocyte-derived microglia-like cells (MDMi) highlighting the need for standardization across protocols [34].

The aim of this study was to characterize and optimize to a certain extent the cultures of MDMi. We have used the protocol by Ohgidani et al. [29] to conduct 49 independent cultures. We summarize here our findings on culture conditions, microglial markers, phagocytosis of α-synuclein aggregates, responses to LPS, and susceptibility to siRNA-mediated inhibition. A report with similar aims studying other microglial responses has been recently published, and it has shown that this model mimics many features of human microglia [35]. These studies are necessary to improve the protocol, to more accurately characterize the resulting cells, and to identify the weaknesses and strengths of the model. They are important to establish a consensus protocol that could be used to set up a well-characterized and reproducible model to study human microglia–like cells in health and disease.

## Methods

### Monocyte-Derived Microglia-Like Cultures

**Fig. 1:**
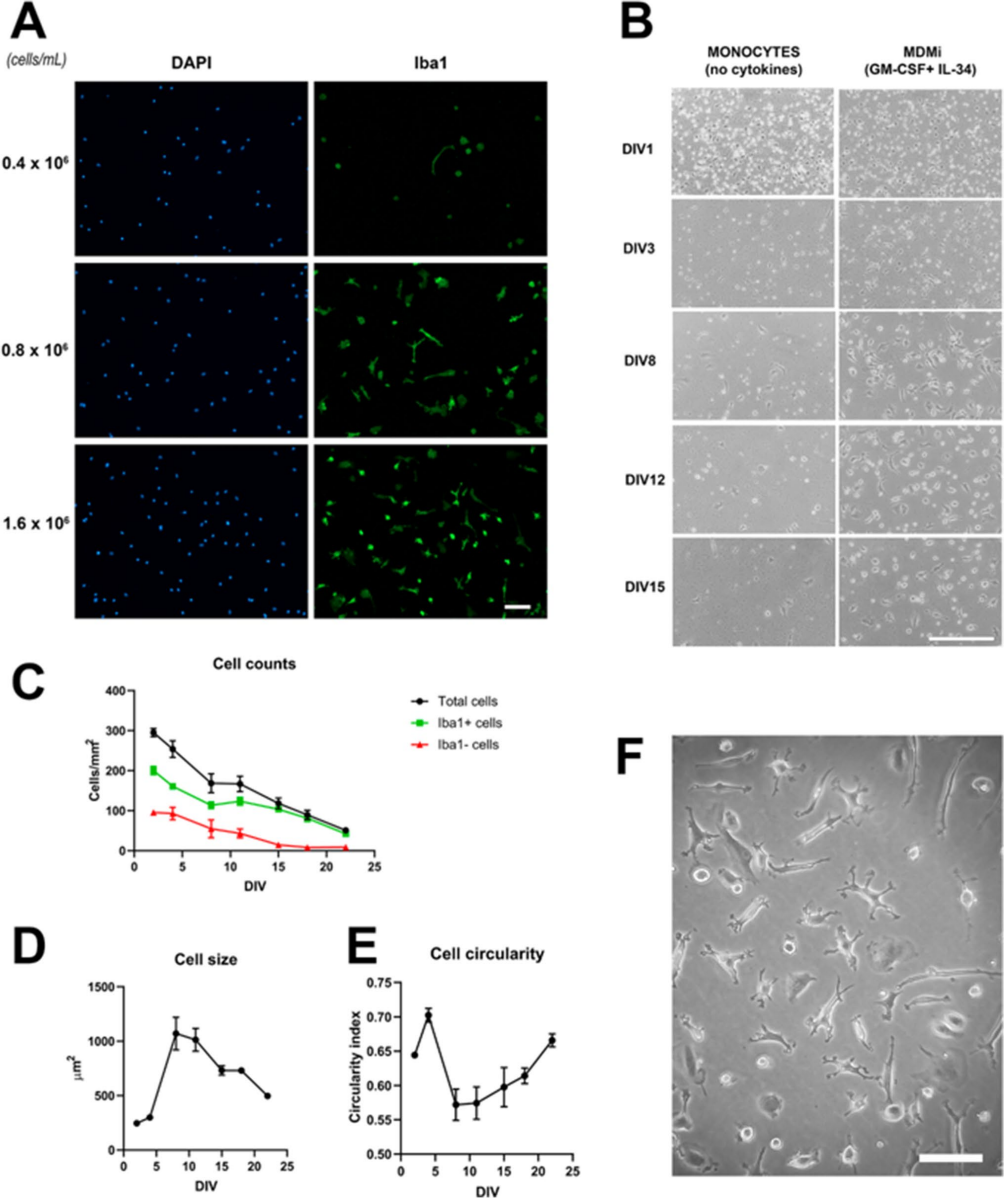
**A** PBMCs were seeded at 0.4, 0.8, and 1.6 × 10^6^ cells/mL, differentiated into microglia-like cells with GM-CSF and IL-34 for 12 days, and stained with DAPI to reveal nuclei (blue) and with the microglial marker Iba1 (green). Note that seeding at the originally reported density of 0.4 × 10^5^ cells/mL resulted in our hands, in a paucity of cells. Images are representative of 4 independent cell culture preparations. Magnification bar, 100 µm. **B** Phase contrast images of PBMCs cultured in the absence or presence of GM-CSF and IL-34 for 1, 3, 8, 12, and 15 days in vitro (DIV). In the absence of cytokines, cells do not differentiate and die, whereas in the presence of GM-CSF and IL-34, many cells survive and show morphological differentiation. Images are representative of 2 independent cell culture preparations. Magnification bar, 250 µm. **C**–**E** Time-course analysis (DIV 2, 4, 8, 11, 15, 18, and 22) in microglia-like cultures of: **C** number of total cells (DAPI-stained), Iba1-positive cells and Iba1-negative cells; **D** cell size, expressed as µm^2^, and **E** cell circularity, expressed as circularity index 4π × (area/perimeter^2^), ranging from 0 to 1, 1 being a perfect circle. Data show mean ± SEM from estimates obtained in 3 wells from a single cell culture preparation. In total, 1413 cells were analyzed. **F** Representative image of microglia-like cells with ramified morphology. Magnification bar, 100 µm

**Fig. 2:**
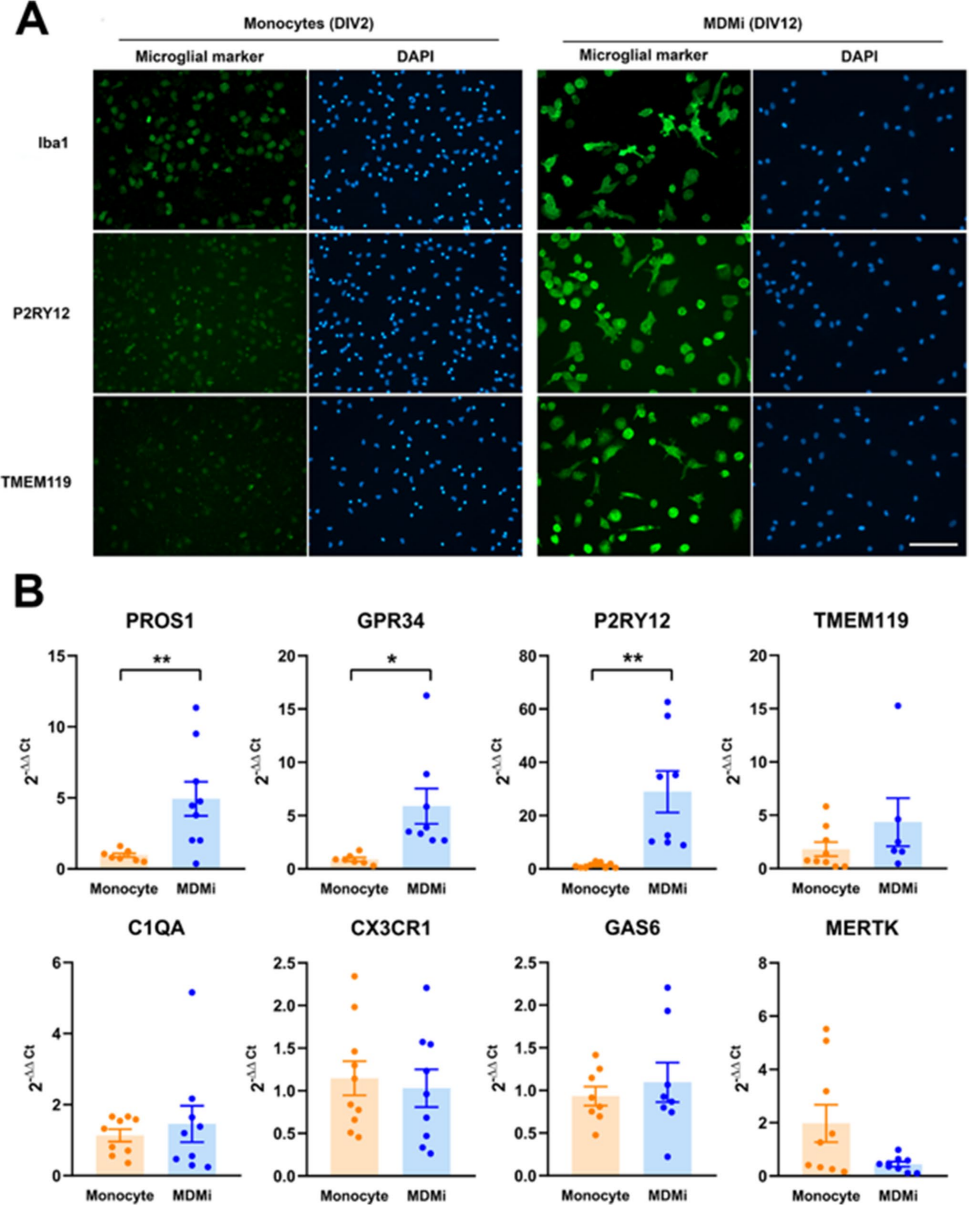
**A** Monocytes and MDMi were immunostained for the microglial markers Iba1, P2RY12, and TMEM119. Note the increase in the immunostaining of the three markers in MDMi when compared with monocytes. Nuclei were counterstained with DAPI. Magnification bar, 100 µm. **B** The mRNA levels of 8 microglial genes were analyzed by qRT-PCR in monocytes (PBMCs cultured for 24 h) and MDMi (PBMCs cultured for 12 days with GM-CSF and IL-34). Data are expressed as 2^−ΔΔCt^ with β-actin and ribosomal protein S18 as reference genes, and monocytes as the control condition. Bars show mean ± SEM of 6–10 independent cell culture preparations. *p < 0.05, **p < 0.01, Student *t*-test. Normality was assessed by the Shapiro–Wilk test. All sets of data showed a normal distribution except GPR34 and C1QA values in MDMi cells, MERTK values in monocytes, and TMEM119 values in both monocytes and MDMI cells. Consequently, GPR34, C1QA, TMEM119, and MERTK means were compared with Mann–Whitney non-parametric test

**Fig. 3:**
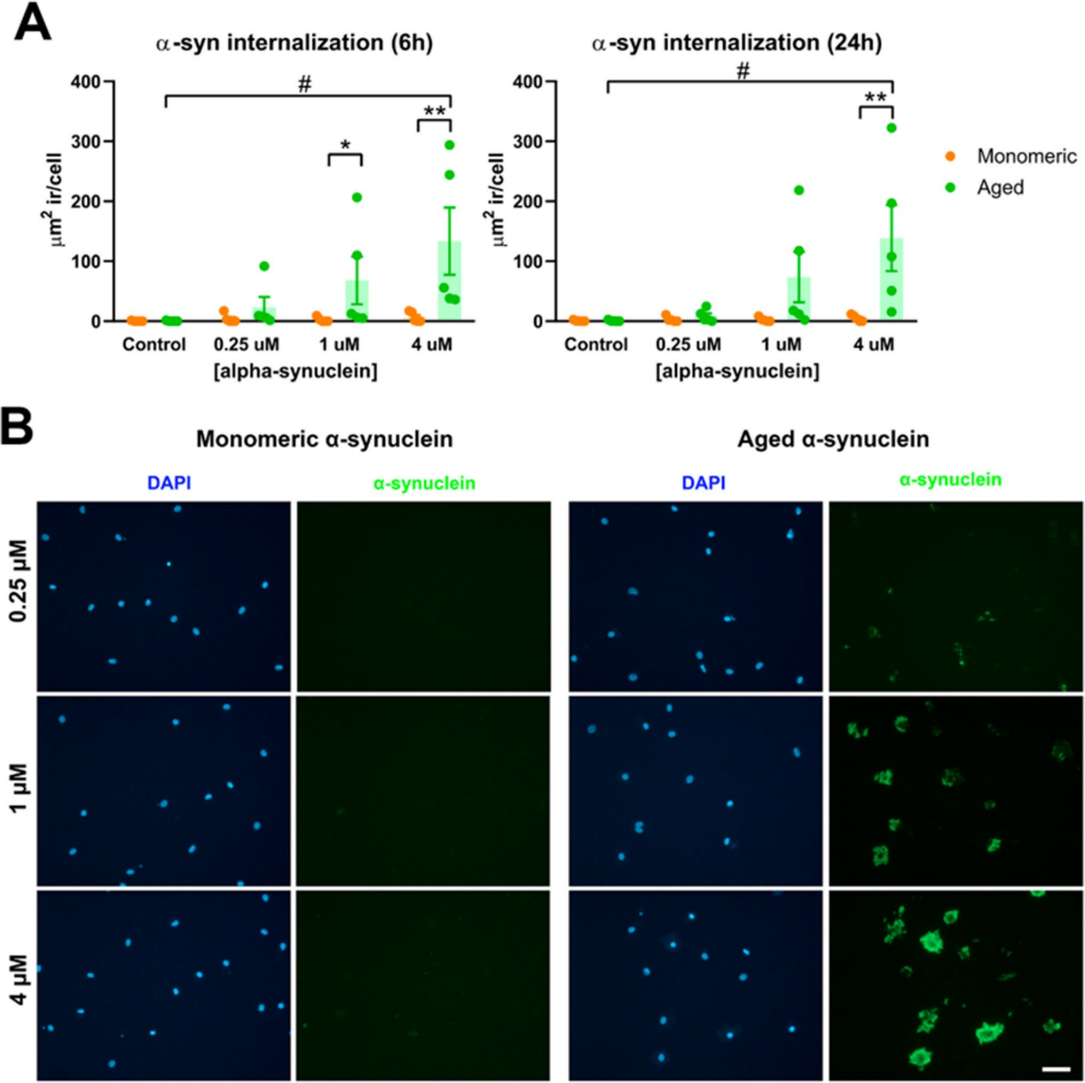
**A** MDMi were incubated for 6 h and 24 h with monomeric or aged α-synuclein at 0.25 µM, 1 µM, and 4 µM. After fixation, α-synuclein was immunostained and the area of intracellular α-synuclein immunostaining was quantified. The graphs show the quantification of intracellular α-synuclein expressed as µm^2^ immunoreactivity per cell. Bars show the mean ± SEM of 5 independent cell culture preparations. Two-way ANOVA revealed a significant effect of α-synuclein concentration and α-synuclein aggregation state, both at 6 h and 24 h. However, since we could not assume that residuals were normally distributed (Shapiro–Wilk test), we compared the groups by non-parametric tests. Kruskal–Wallis test followed by Dunn’s multiple comparison test was used to compare values from different α-synuclein concentrations with control (*p < 0.05, **p < 0.01), and Mann–Whitney test to compare monomeric vs aged α-synuclein at a given concentration (^#^p < 0.05). Bonferroni correction was used to adjust for multiple comparisons. **B** Representative images of α-synuclein immunoreactivity in MDMi treated for 24 h with monomeric and aged α-synuclein at 0.25 µM, 1 µM, and 4 µM. Nuclei were stained with DAPI (blue). Note the absence of intracellular α-synuclein immunostaining in cells treated with monomeric α-synuclein and the concentration-dependent increase in cells treated with aged (fibrillar) α-synuclein. Magnification bar, 50 µm

**Fig. 4:**
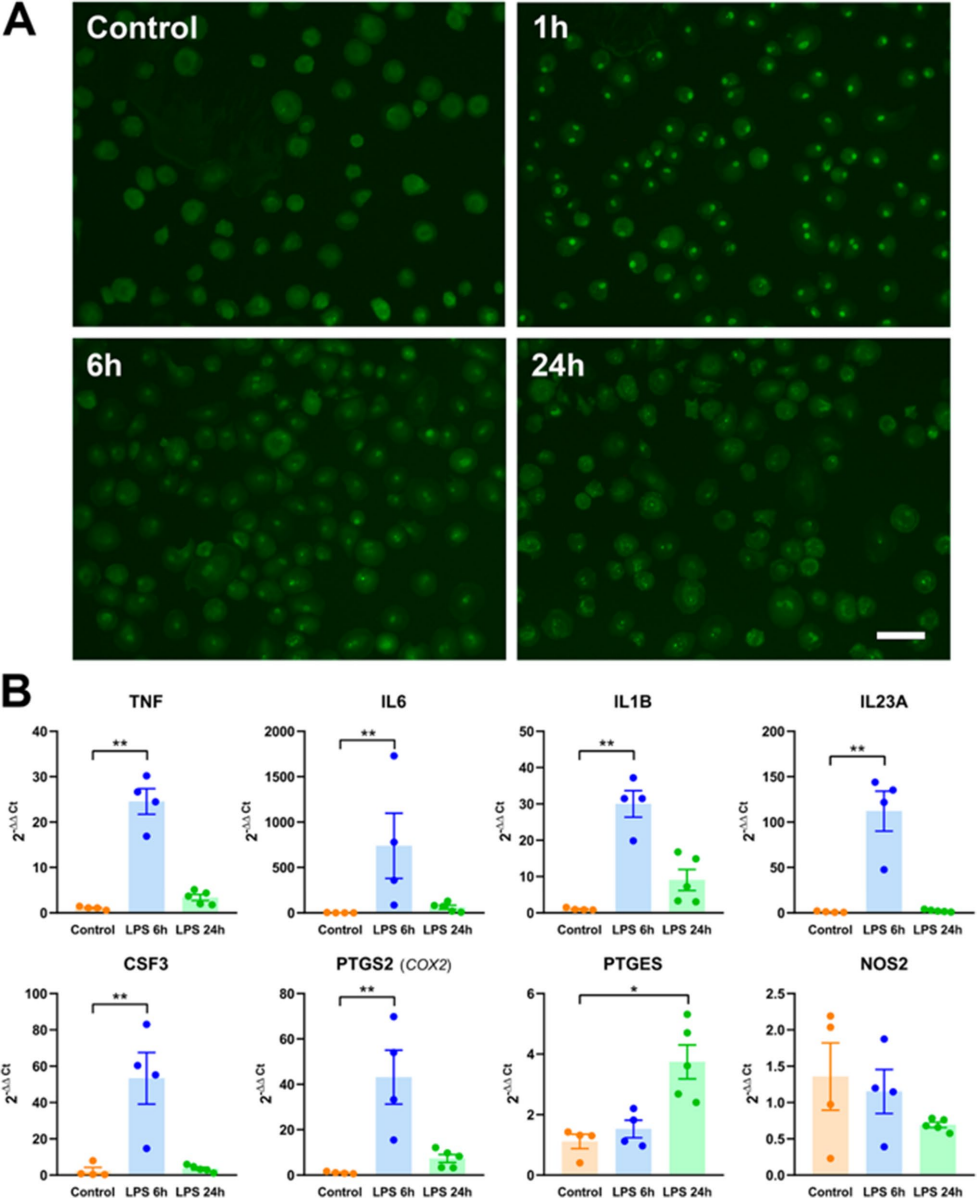
**A** MDMi were treated with vehicle (control) or LPS 100 ng/mL for 1 h, 6 h, and 24 h and immunostained with antibodies against the p65 subunit of NF-kappaB. Whereas in untreated cells, p65 immunoreactivity is cytosolic; LPS induces a prominent nuclear localization of p65 after 1 h, which declines over time. Magnification bar, 50 µm. **B** MDMi were treated with vehicle (control) or LPS 100 ng/mL for 6 h and 24 h, and the mRNA levels of 8 proinflammatory genes were analyzed by qRT-PCR. Data are expressed as 2^−ΔΔCt^ with β-actin and ribosomal protein S18 as reference genes, and vehicle as the control condition. Bars show mean ± SEM of 4–5 independent cell culture preparations. *p < 0.05, **p < 0.01, vs control. One-way ANOVA followed by Dunnett’s multiple comparison test. Normality was assessed by the Shapiro–Wilk test

**Fig. 5:**
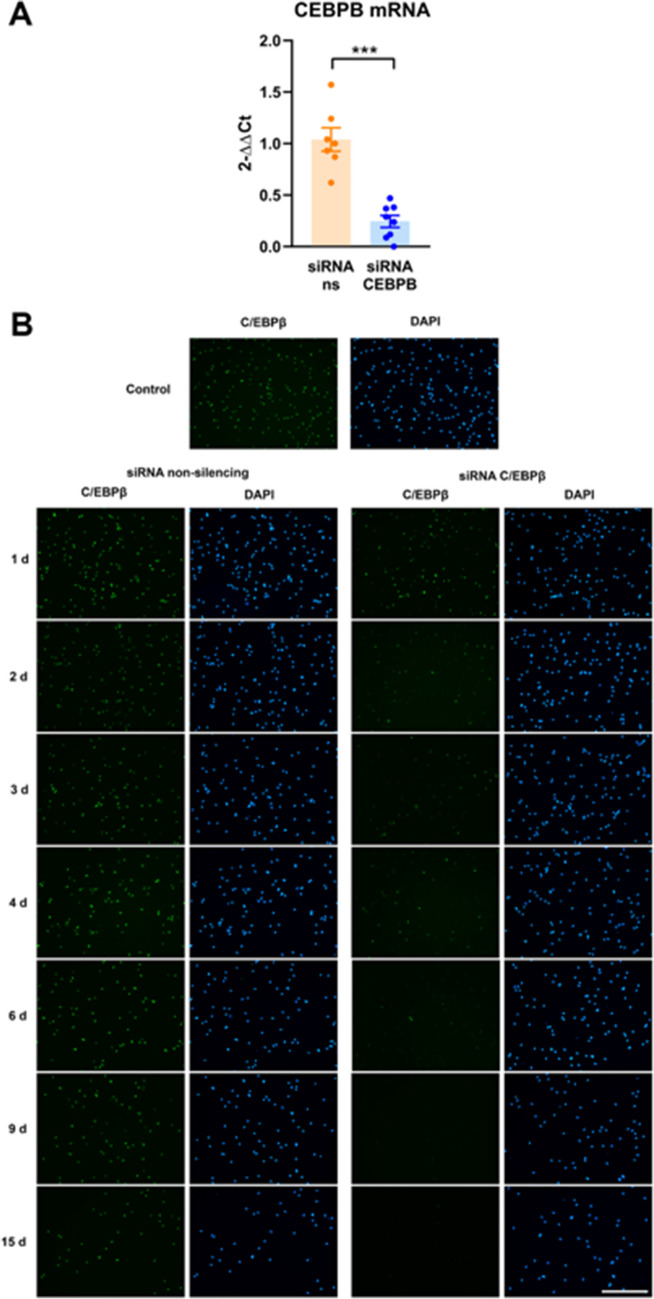
**A** MDMi were treated for 48 h with 25 nM siRNAs targeting C/EBPβ or non-silencing siRNAs (ns). The mRNA levels of C/EBPβ were analyzed by qRT-PCR. Data are expressed as 2^−ΔΔCt^ with β-actin and ribosomal protein S18 as reference genes, and non-silencing siRNAs as the control condition. Bars show mean ± SEM of 7–8 independent cell culture preparations. ***p < 0.001, Student *t*-test. Normality was assessed by the Shapiro–Wilk test. **B** MDMi were treated with 25 nM non-silencing siRNAs or siRNAs targeting C/EBPβ, for various times between 24 h and 15 days. Cells were then immunostained for C/EBPβ (green) and nuclei were counterstained with DAPI (blue). C/EBPβ immunoreactivity is present in virtually all cells in the control condition (untreated) or treated with non-silencing siRNA at all time points. In contrast, siRNAs targeting C/EBPβ cause a time-dependent reduction in the intensity of C/EBPβ immunoreactivity and in the number of C/EBPβ-positive cells. Magnification bar, 100 µm

**Fig. 6:**
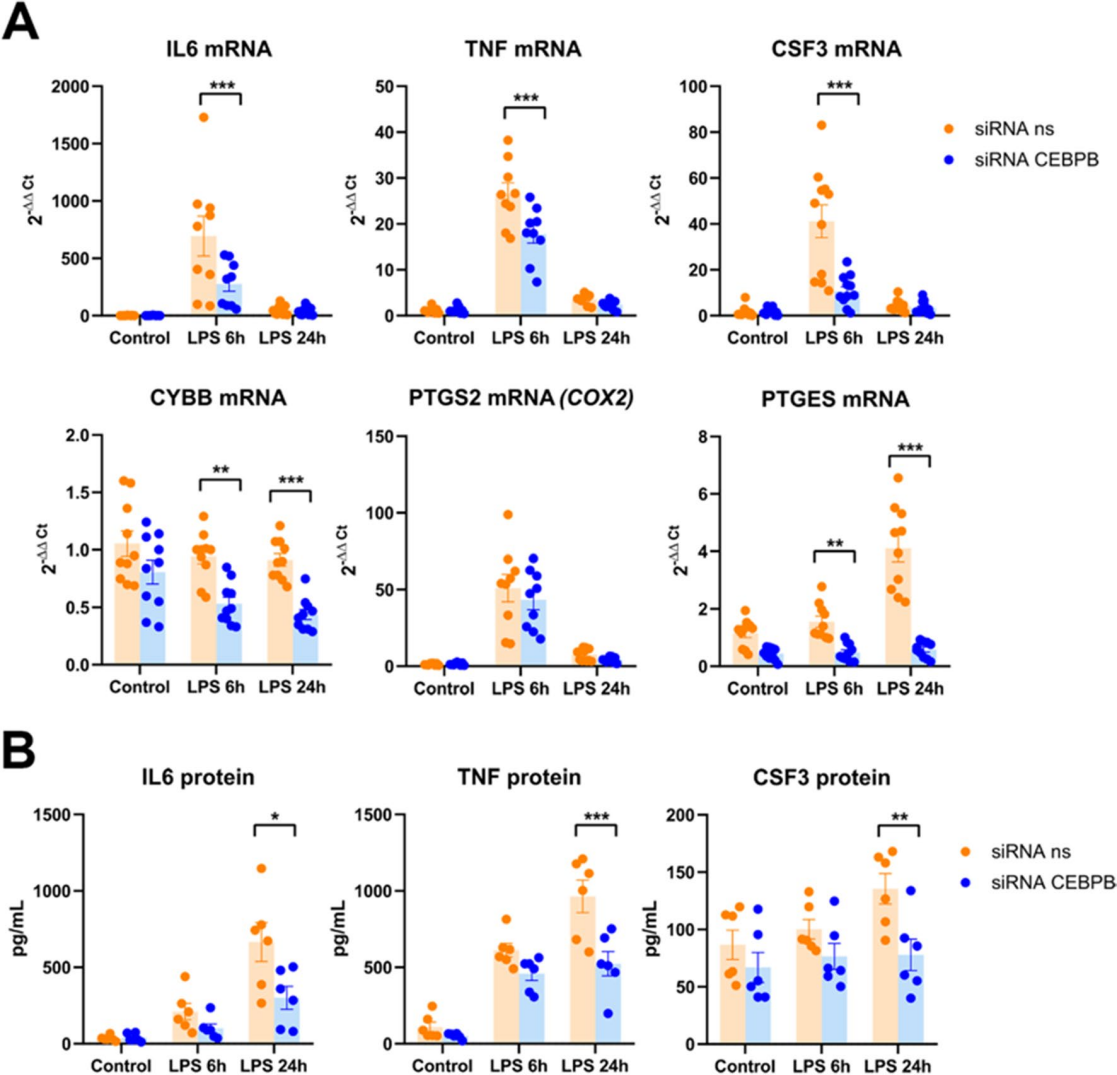
MDMi were pretreated with 25 nM non-silencing siRNAs (siRNA ns) or siRNAs targeting C/EBPβ for 48 h before treatment with vehicle (Control), LPS 6 h or LPS 24 h. **A** mRNAs of several proinflammatory genes were analyzed by qRT-PCR. Data are expressed as 2^−ΔΔCt^ with β-actin and ribosomal protein S18 as reference genes, and vehicle/non-silencing siRNAs as the control condition. Bars show mean ± SEM of 8–11 independent experiments. *p < 0.05, **p < 0.01, ***p < 0.001 vs respective non-silencing siRNA condition. Two-way ANOVA followed by Sidak’s multiple comparison test. Normality was assessed by the Shapiro–Wilk test. **B** Proinflammatory mediators IL6, TNF, and CSF3 were analyzed in the conditioned medium by ELISA. Data are expressed as pg/mL. Bars show mean ± SEM of 6 independent cell culture preparations. *p < 0.05, **p < 0.01, *** *p* < 0.001 vs respective non-silencing siRNA condition. Two-way ANOVA followed by Sidak’s multiple comparison test. Normality was assessed by the Shapiro–Wilk test

This protocol is adapted from Ohgidani et al. [29]. Peripheral blood was obtained from 49 healthy volunteers (29 females, 20 males; mean age 36.7 years (SD = 16.2), range 23–68 years). All participants provided informed consent and the study was approved by the Ethics Committee of the Hospital Clínic de Barcelona (Ref. HCB/2021/0349). Sixty milliliters of peripheral blood from each donation were collected into EDTA tubes (367525, BD). Peripheral blood mononuclear cells (PBMCs) were isolated by density gradient centrifugation (400 g, 30 min) using Histopaque-1077 (10,771, Sigma-Aldrich) in a 1:1 proportion. After centrifugation, the interphase containing the PBMCs, approximately 3 mL per 20 mL of blood, was recovered. After two washing steps (200 g, 10 min) in culture medium (RPMI-1640 GLUTAMAX (61870–044, Invitrogen), penicillin–streptomycin (100 U/mL and 100 µg/mL respectively; Life Technologies), Fungizone (0.25 µg/mL; Life Technologies) 10% Fetal Bovine Serum (FBS; Life Technologies)), PBMCs were resuspended in culture medium, counted and plated at a density of 1.6 × 10^6^ cells/mL onto 24- or 48-well plates (400 µL and 200 µL per well, respectively), and cultured at 37 °C, 5% CO2. In most cases, PBMCs were seeded onto uncoated plates, but poly-l-lysine coating (10 µg/mL) was used for comparison in some experiments. After overnight incubation, adherent cells were collected, corresponding to monocytes, or the medium was aspirated and replaced by fresh culture medium containing rhGM-CSF (10 ng/mL; RYD-7954-GM, R&D Systems) and rhIL-34 (100 ng/mL; 200–34, Peprotech) to prepare MDMi. Cultures were used at day-in-vitro (DIV) 12–16 unless otherwise indicated. In four experiments, isolated PBMCs were frozen according to a published protocol [36]. More than 2 months after freezing, PBMCs were thawed and seeded for MDMI differentiation following the protocol described from freshly isolated PBMCs. A total of 49 independent cultures were performed, and the data here presented in Figs. 1, 2, 3, 4, 5, and 6 was obtained from 40 out of the 49 cultures performed. A detailed working protocol is included as Supplementary File 1.

### Treatments

#### Lipopolysaccharide (LPS)

Cultures were treated with LPS (*E. coli* 026:B6 serotype, L2654, Sigma-Aldrich) 100 ng/mL (diluted in RPMI-1640 GLUTAMAX from a 1 mg/mL stock) or vehicle, for 6 or 24 h.

#### α-Synuclein

Cells were treated with monomeric or aged α-synuclein (see aging procedure below) at three concentrations (0.25, 1, and 4 µM) or vehicle, for 6 and 24 h.

#### siRNAs

siRNA transfection was carried out following indications from the supplier (Dharmacon). Pools of 4 siRNAs (SMARTPool) targeting CEBPB (M-006423–03-0005, Dharmacon) and non-targeting (D-001206–13-05, Dharmacon) were used. Briefly, for each condition, 10 µL of the siRNA stock (5 mM) + 190 µL of RPMI-1640 GLUTAMAX and 4 µL of reagent 4 + 196 µL of RPMI-1640 GLUTAMAX were mixed in two tubes. After a 5-min incubation, the content of both tubes was mixed and incubated for 20 min. Then, 1.6 mL of RPMI-1640 GLUTAMAX was added to obtain a 25 nM siRNA solution. Finally, the medium was aspirated and replaced by the siRNA solution. Cells were treated with siRNA for 48 h except for the time-course experiment shown in Fig. 5B where treatment spanned from 24 h to 15 days.

### Aging of α-Synuclein

One milligram of lyophilized α-synuclein (rPeptide, S-1001–2) was reconstituted in 1 mL of sterile phosphate buffered saline (PBS) and split into two 500 µL fractions to keep half of the total volume as monomeric α-synuclein and to submit the rest to the aging protocol. The aging protocol consisted of incubating α-synuclein solution for 14 days at 37 °C in constant agitation in a shaker at 250 rpm. Both monomeric and aged protein stocks were stored at − 20 °C [37, 38]. Amyloid fibril formation in aged α-synuclein samples was confirmed by electron microscopy (Supplementary file 2).

### Immunocytochemistry and Image Analysis

Immunocytochemistry was performed in cultures fixed with 4% paraformaldehyde (20 min, RT; Sigma). Fixed cells were washed with PBS (5 min; Sigma) and stored in PBSt (PBS + 0.1% thimerosal (Sigma)) at 4 °C. After methanol (PanReac) permeabilization (7 min, − 20 °C), cells were washed in PBS and incubated overnight at 4 °C in primary antibody (Table 1) diluted in immunobuffer (PBSt + 7% normal goat serum (Life Technologies)). After overnight incubation, cells were washed in PBS and incubated for 1 h at RT in secondary antibody (Table 1) with 4′,6-diamidine-2′-phenylindole dihydrochloride (DAPI, 5 µg/mL; Sigma D9542). Cells were washed with PBS and stored in PBSt at 4 °C light-protected. An epifluorescence microscope (Olympus IX70, Olympus, Okoya, Japan) coupled with a digital camera (CC-12, Olympus Soft Imaging Solutions GmbH, Hamburg, Germany) was used for the observation and acquisition of images. Cell nuclei counting and morphometric analysis (cell size and circularity) were carried out using ImageJ (1.52a, NIH) on microscopy images obtained at × 4 and × 20 magnification, respectively. The circularity index was calculated with the formula 4π × (area/perimeter^2^), ranging from 0 to 1, a perfect circle. To calculate the area occupied by intracellular α-synuclein, cell nuclei were counted on each image, and the area occupied by α-synuclein was estimated using ImageJ. The area occupied by α-synuclein per microglial cell was calculated by dividing the area occupied by α-synuclein by the nuclei number.

**Table 1:** List of antibodies

Type	Antigen	Dilution	Reference	Supplier
Primary antibodies				
Rabbit polyclonal	Iba1	1:500	019–19741	Wako
Rabbit polyclonal	P2RY12	1:1000	HPA014518	Sigma-Aldrich
Rabbit polyclonal	TMEM119	1:500	HPA051870	Sigma-Aldrich
Mouse monoclonal	α-Synuclein	1:200	NCL-LASYN	Novocastra
Mouse monoclonal	NF-kB p65	1:100	(F-6): sc-8008	Santa Cruz
Mouse monoclonal	C/EBPβ	1:500	(H-7) sc-7962	Santa Cruz
Secondary antibodies				
Alexa 488-conjugated Goat polyclonal	Mouse IgG	1:1000	A11017	Life Technologies
Alexa 488-conjugated Goat polyclonal	Rabbit IgG	1:1000	A11070	Life Technologies

### Quantitative Real-Time PCR (qRT-PCR)

The medium was removed from 4 to 6 wells of a 24-well plate, and 350 µL of lysis solution (lysis buffer (PureLink RNA Micro Kit, Invitrogen) and β-mercaptoethanol (Sigma)), in a 100:1 proportion, were passed through the wells, pipetting up and down and scratching with the pipette tip. The recovered 350 µL was stored at − 80 °C until RNA extraction. Cell lysates were passed through a 21G needle and 350 µL of 70% ethanol (PanReac) were added. From the resulting 700 µL, RNA was extracted using a commercial kit (PureLink RNA Micro Kit, Invitrogen). The RNA concentration in each sample was determined with Nanodrop One (ND-1000, Thermo Scientific). The RNA yield of 6 wells from 24-well plates was 481.5 ± 157.0 ng/µL (*n* = 15) and 436.2 ± 139.6 ng/µL (*n* = 4) when MDMIs were prepared from freshly isolated PBMCs, or frozen and thawed PBMCs, respectively. Samples were stored at -80 °C until RT-qPCR. RNA was reverse transcribed with random primers using Transcriptor Reverse Transcriptase (Roche Diagnostics) to obtain cDNA by using qScriptTM cDNA Synthesis Kit (Quanta Biosciences, Ref. 95,047) according to manufacturer’s instructions. To perform the retrotranscription, samples were diluted in autoclaved Milli-Q water for a final RNA concentration of 10 ng/µL. Fifteen microliter of the sample was mixed with 5 µL of a mix solution (4 µL of qScript Reaction MIX and 1 µL of qScript Reverse Transcriptase (qScript cDNA Synthesis Kit, QUANTA BIOSCIENCES)). The reverse transcription was performed using a thermal cycler (MJ Mini, BioRad) under the following protocol: 25 °C for 5 min, 42 °C for 30 min, and 85 °C for 5 min. Samples were diluted in autoclaved Milli-Q water for a final concentration of 1 ng/µL.

For the qRT-PCR, a mix solution was prepared for each gene, consisting of 0.45 µL of Fwd primer + 0.45 µL of Rev primer + 3.6 µL of Milli-Q water + 7.5 µL of SYBR Green (BioRad) (see Table 2 for primers). Three microliters of the cDNA sample and 12 µL of the corresponding mix solution were loaded in duplicates in the RT-PCR plate. For each gene, a no template control (3 µL of Milli-Q water) was also added. Plates were covered with a plate sealer and centrifuged (1000 rpm, 5 s). The qRT-PCR was performed (15 s at 95 °C, 30 s at 60 °C, 15 s at 72 °C; 39 cycles) in an iCycler MyIQ apparatus (Bio-Rad Laboratories). ACTB (β-actin) and RPS18 (ribosomal protein S18) were used as reference genes. Results were processed with the BioRad CFX manager. qRT-PCR data were analyzed using the 2^−ΔΔCt^ method [39].

**Table 2:** List of primers used for qRT-PCR

Gene	Sequence	
RPS18	Forward:	5′- GAT GGG CGG CGG AAA ATA GC -3′
	Reverse:	5′- GAG TTC TCC CGC CCT CTT GG -3′
ACTB	Forward:	5′- GCC TCG CCT TTG CCG ATC C -3′
	Reverse:	5′- CAC ATG CCG GAG CCG TTG TC -3′
PROS1	Forward:	5′- TGC TGG CGT GTC TCC TCC TA -3′
	Reverse:	5′- CAG TTC TTC GAT GCA TTC TCT TTC A -3′
GPR34	Forward:	5′- CTC CCA CAG AAT GCG CTT TAT A -3′
	Reverse:	5′- CAA CCA GTC CCA CGA TGA AAA -3′
P2RY12	Forward:	5′- CAA GCC GTC GAC AAC CTC ACC TC -3′
	Reverse:	5′- TCT CGG CTG CCT GTT GGT CAG AA -3′
TMEM119	Forward:	5′- GAG GCA CTC TAC GGA AAC -3′
	Reverse:	5′- CGG GAG AAT CGC TTG AAC -3′
C1QA	Forward:	5′- TCT GCA CTG TAC CCG GCT A -3′
	Reverse:	5′- CCC TGG TAA ATG TGA CCC TTT T -3′
CX3CR1	Forward:	5′- TGC TGT GCT GTG CCC AAG TT -3′
	Reverse:	5′- CTC CCC AGG TTG GCA GTA GC -3′
GAS6	Forward:	5′- CAT CAA CAA GTA TGG GTC TCC GT -3′
	Reverse:	5′- GTT CTC CTG GCT GCA TTC GTT GA -3′
MERTK	Forward:	5′- ACT TCA GCC ACC CAA ATG TC-3′
	Reverse:	5′- GGG CAA TAT CCA CCA TGA AC -3′
CEBPB	Forward:	5′- TTT GGC ACT GGG GCA CTT GG -3′
	Reverse:	5′- AAA TAA CAC CAC GGG CGG GA -3′
NOS2	Forward:	5′- CTT TGA TGA GGG GAC TGG GCA -3′
	Reverse:	5′- CCG GGG TAA GGA CAG TCA AAC C -3′
IL6	Forward:	5′- GCC AGA GCT GTG CAG ATG AGT -3′
	Reverse:	5′- AGC AGG CTG GCA TTT GTG GT -3′
IL1B	Forward:	5′- CTC TTC GAG GCA CAA GGC ACA -3′
	Reverse:	5′- ATT TCA CTG GCG AGC TCA GGT -3′
TNF	Forward:	5′- CGA ACC CCG AGT GAC AAG CC -3′
	Reverse:	5′- CCA TTG GCC AGG AGG GCA TT -3′
PTGS2 (= COX2)	Forward:	5′- CGA GGG CCA GCT TTC ACC AA -3′
	Reverse:	5′- AGG CGC AGT TTA CGC TGT CT -3′
IL23A	Forward:	5′- TGA GAA GCT GCT AGG ATC GG -3′
	Reverse:	5′- ACT GAG GCT TGG AAT CTG CT -3′
CYBB	Forward:	5′- GAA CTG GGC TGT GAA TGA GGG G -3′
	Reverse:	5′- AGT GCC AGT GCT GAC CCA AGA -3′
CSF3	Forward:	5′- GAG CAA GTG AGG AAG ATC CAG -3′
	Reverse:	5′- CAG CTT GTA GGT GGC ACA CA -3′
PTGES	Forward:	5′- CCC CCA GTA TTG CAG GAG CG -3′
	Reverse:	5′- GGA AGT GCA TCC AGG CGA CA -3′

### ELISA

The protein levels of three proinflammatory mediators were analyzed in the conditioned medium of MDMi treated with LPS and siRNAs. The conditioned media were collected 6 h and 24 h after treatments. IL-6 (#900-TM16, Peprotech), TNFα (#900-TM25, Peprotech), and CSF3 (#900-K77, Peprotech) were quantified by Sandwich ELISA kits following the manufacturer’s instructions.

### Statistical Analyses

Results are presented as mean ± SEM. Statistical analyses were performed using GraphPad Prism 4.02 (GraphPad Software Inc, La Jolla, USA). Normality was assessed by the Shapiro–Wilk test. If data passed the normality test, statistical significance was evaluated with parametric tests: unpaired Student two-tailed *t*-test for comparing two groups, one-way ANOVA followed by Dunnett’s multiple comparison test for comparing more than two groups involving one factor, and two-way ANOVA followed by Sidak’s multiple comparison test for comparing more than two groups involving two factors. When data did not pass the Shapiro–Wilk normality test, statistical significance was evaluated with non-parametric tests. This occurred in the data shown in Fig. 3A. This was a two-factor experiment with 3 α-synuclein concentrations and two α-synuclein states. In this case, the Kruskal–Wallis test was used to compare the values from different α-synuclein concentrations with control, and the Mann–Whitney test to compare monomeric vs aged α-synuclein at a given concentration, with Bonferroni correction to adjust for multiple comparisons. A confidence interval of 0.95 was set, and therefore, *p*-values lower than 0.05 were regarded as statistically significant.

## Results

Observations from pilot experiments using the protocol described by Ohgidani et al. [29] to differentiate monocytes into microglia-like cells revealed a low cell density after 10–15 DIV. Such cell density, based on our experience with primary mouse microglia, would result in low RNA and protein levels for subsequent qRT-PCR, Western, or ELISA studies. On the other hand, too scattered cells can be a detrimental factor for cell survival [40]. To overcome these limitations, we compared the progression of cultures seeded at the original density of 400,000 cells/mL (× 1), with higher densities of 800,000 cells/mL (× 2) and 1,600,000 cells/mL (× 4). In four independent cell culture preparations, we observed that seeding at 1,600,000 cells/mL resulted in optimal final cell densities for the following studies (Fig. 1A). Quantification of one of these experiments resulted in final cell densities of 118.7 ± 9.4 cells/mm^2^, 130.4 ± 3.2 cells/mm^2^, and 226.9 ± 5.8 cells/mm^2^, (DIV12, *n* = 3 wells per condition) for seeding densities of × 1, × 2, and × 4, respectively. Based on these observations, we used a seeding density of 1,600,000 cells/mL in all subsequent experiments. Since PBMC yield was 49.8 ± 3.6 × 10^6^ cells per experiment, this resulted in 31.1 ± 2.2 mL of seeding solution per experiment.

The original publication by Ohgidani et al. [29] does not mention whether cells were seeded onto coated or uncoated culture wells. Comparison of cultures seeded on uncoated vs poly-l-lysine-coated wells showed a higher cell density in cultures growing on uncoated wells (180.7 ± 0.6 cells/mm^2^ (uncoated) vs 94.2 ± 7.4 cells/mm^2^ (coated), DIV12, *n* = 3 wells per condition) and no marked differences in terms of cell size (791,150 ± 108,107 µm^2^ (uncoated) vs 1,088,349 ± 230,932 µm^2^ (coated), DIV12, *n* = 3 wells per condition) or cell circularity (0.529 ± 0.016 (uncoated) vs 0.564 ± 0.020 (coated), DIV12, *n* = 3 wells per condition). Based on these observations, we plated monocytes onto uncoated plastic wells in all subsequent experiments. This agrees with our experience with primary mouse microglial cultures showing that microglial cells attach and grow well on plastic surfaces [41].

The differentiation protocol used here relies on the use of the cytokines GM-CSF and IL-34 [29]. To confirm the effectiveness of this treatment we compared the progression of monocytes cultured in the presence or absence of these cytokines. As shown in Fig. 1B, monocytes die rapidly when cultured in the absence of GM-CSF and IL-34, whereas in the presence of the cytokines, a clear pro-survival and differentiating effect is observed. We quantified this effect in terms of cell density and cell morphology. As seen in Fig. 1, in the presence of GM-CSF and IL-34, cell density progressively decreases (Fig. 1C), cell size markedly increases during the first 8 days in vitro (Fig. 1D), and then both parameters stabilize between DIV8 and DIV15. In parallel, cell circularity decreases during the first 8 days in vitro, and it reaches a plateau between DIV8 and DIV15 (Fig. 1E). This decrease in cell circularity probably reflects the differentiation of round monocytes into ramified microglia-like cells. These data suggest that in terms of morphology and cell density, experiments should be performed between DIV8 and DIV15. At earlier time points, cells show a monocytic morphology, round and small, and at later time points, viability and morphological differentiation appear to decrease. Based on these data, we performed all subsequent experiments between DIV12 and DIV15.

To ascertain the microglial phenotype of GM-CSF/IL-34-differentiated monocytes, we compared the expression of microglial markers in monocytes (DIV2) and MDMi (DIV12) by immunocytochemistry and qRT-PCR (Fig. 2). Immunocytochemistry experiments showed a marked increase in the immunostaining of the microglial markers Iba1, P2RY12, and TMEM119 in MDMi when compared with monocytes (Fig. 2A). qRT-PCR experiments showed a significant upregulation of the microglial markers PROS1, GPR34, and P2RY12 in MDMi (Fig. 2B). In contrast, the levels of the markers C1QA, CX3CR1, GAS6, TMEM119, and MERTK were not significantly different between monocytes and MDMi, although a tendency for an increase in TMEM119 in MDMi was observed (Fig. 2B). These findings indicate that the protein and mRNA expression profile of MDMi cells show many features of a microglial phenotype. However, there is still room for optimization of the differentiation protocol, to fully reproduce the molecular profile of human microglial cells in a physiological context.

A defining feature of microglial cells is their high phagocytic ability. Microglia can phagocytose apoptotic cells, myelin debris, dystrophic neurites, unwanted synapses, pathogens, or abnormal bodies. Of particular importance in human disease is the microglial phagocytosis of abnormal protein aggregates such as Aβ and α-synuclein aggregates found in Alzheimer’s and Parkinson’s disease, respectively [8, 42]. Dysregulated phagocytosis can result in the accumulation of such abnormal proteins and participate in pathogenesis. We, therefore, tested the phagocytosis of aged α-synuclein fibrils vs monomeric α-synuclein by microglia-like cells. Immunocytochemistry against α-synuclein of MDMi cultures treated for 6 h or 24 h with different concentrations (0.25, 1, and 4 µM) of monomeric and aged α-synuclein showed strong phagocytosis of aged α-synuclein in a concentration-dependent manner, both at 6 h and 24 h (Fig. 3). In contrast, internalization of monomeric soluble α-synuclein was not observed (Fig. 3). These results show that this is a suitable model to study phagocytosis of abnormal protein aggregates by human microglia–like cells.

Another important feature of microglial cells, though not cell type–specific, is their ability to respond to LPS exposure with the activation of the transcription factor NFkappaB and the subsequent upregulation of proinflammatory genes. To confirm these responses, MDMi were treated with LPS (100 ng/mL); NFkappaB activation was assessed by p65 immunocytochemistry and upregulation of proinflammatory mediators by qRT-PCR for the genes TNF, IL6, IL1B, PTGS2 (= COX2), PTGES, NOS2, IL23A, and CSF3. LPS induced a rapid and transient activation of NFkappaB. As shown in Fig. 4A, the NFkappaB subunit p65 is mainly cytosolic in untreated MDMi, and LPS induces its nuclear translocation, which is maximal at 1 h and then declines at 6 h and even further at 2 4h. Most of the proinflammatory genes analyzed, TNF, IL6, IL1B, IL23A, and CSF3, showed a marked upregulation at the mRNA level 6 h after LPS treatment (Fig. 4B). This upregulation was transient, as mRNA levels returned to control levels at 24 h. PTGES was the only case among the genes analyzed showing a delayed upregulation of mRNA levels at 24 h, and not at 6 h, and NOS2 was the only gene not being upregulated by LPS (Fig. 4B). Altogether, these findings indicate that this is a suitable model to study proinflammatory responses of human microglia–like cells.

We next were interested in studying whether MDMi are amenable to RNA silencing by siRNAs. To test this, we studied the siRNA-induced silencing of the transcription factor C/EBPβ, with the official gene name CEBPB. In studies with murine primary glial cultures, we demonstrated that this transcription factor is an important regulator of the pro-inflammatory gene expression program in microglia [5, 7]. qRT-PCR from mRNAs extracted from MDMi treated with siRNAs for 48 h revealed a strong C/EBPβ silencing (76.4% reduction) by siRNAs against C/EBPΒ (Fig. 5A). We then analyzed by immunocytochemistry the effects of siRNAs on C/EBPβ protein levels in MDMi. As shown in Fig. 5B, siRNA treatment resulted in an almost complete disappearance of C/EBPβ immunoreactivity. This decrease was clearly observed 48 h after siRNA treatment, it peaked around 4–6 days, and it was maintained up until the last time-point analyzed, which was day 15. These results demonstrate that MDMi express the essential transcriptional regulator C/EBPβ and that its expression can be long-lastingly targeted with siRNAs.

Finally, we were interested in studying the effects of C/EBPβ silencing on LPS-induced human microglial expression of proinflammatory genes. C/EBPβ inhibition resulted in a significant decrease in the mRNA levels of key pro-inflammatory genes such as IL6, TNF, CSF3, CYBB, and PTGES (Fig. 6A). The expression of the prostaglandin-synthesis enzyme COX2 was not affected. We then used ELISA to study whether the decreased mRNA levels of proinflammatory genes caused by C/EBPβ silencing were strong enough to cause decreased levels of their associated proteins. As shown in Fig. 6B, siRNA C/EBPβ silencing significantly reduced the LPS-induced upregulation in the extracellular levels of IL6, TNF, and CSF3 (Fig. 6B). These results strongly suggest that C/EBPβ is an important regulator of proinflammatory gene expression in activated human microglia–like cells.

## Discussion

To our knowledge, the protocol by Ohgidani and collaborators to prepare microglia-like cell cultures from adult human monocytes [29] has been used to date in eight independent studies [32, 33, 35, 43–47]. While some authors have strictly followed the original protocol, many have introduced modifications in parameters such as the PBMC isolation method, plate coating, or the frequency of medium changes. Our study is part of this ongoing effort aimed at better understanding the protocol and defining the properties of the resulting cells.

### On Technical Issues: PBMC Isolation and Cryopreservation, Coating, Seeding Density, Culture Medium

In this study, PBMCs were isolated with Histopaque density gradient centrifugation, as in the original report and in many subsequent publications, although some authors have used alternative methods to isolate PBMCs such as CD14 beads [44] or Lymphoprep [45]. In all experiments here reported, MDMi were differentiated from freshly isolated PBMCs, although we have used cryopreserved PBMCs with very similar results, and this is the approach used in some studies [32, 45]. In the original report and in most subsequent publications, it was not stated whether PBMCs were seeded on coated or uncoated cell culture plates. We have observed that PBMCs seeded unto uncoated plastic plates differentiate well into microglia-like cells, and the poly-l-lysine coating does not result in an improvement on the final culture. This is consistent with the observation that microglial cells from primary mouse cultures attach and grow well on uncoated plastic surfaces [41]. In recent studies, some authors have used other coating substrates such as Geltrex [32, 35, 44] or Matrigel [45].

In contrast to all previous publications, we used a PBMC cell seeding concentration that was four times higher than that reported by Ohgidani et al. [29]. Seeding at the original concentration resulted in a low density of MDMi at the final stage of the protocol (DIV12-14). This is an important difference that could reflect differences in the cell counting criteria or, more likely, in cell survival in our settings. Despite this, when we qualitatively compared our images with those reported by others [29, 32, 35, 44, 46, 47], the final cell densities were similar suggesting that the final cultures are comparable.

PBMCs were here cultured and differentiated into MDMi using the culture medium (RPMI-1640) and cytokines (10 ng/mL GM-CSF and 100 ng/mL IL-34) described by Ohgidani et al. [29] and used in all subsequent publications. Independently, other authors have described alternative methods using different cocktails of cytokines and growth factors to differentiate iPSCs or monocytes into microglia-like cells [30, 48]. Finally, in this study, we have changed the culture medium at DIV1 and DIV7, as reported by Ohgidani and most subsequent authors. It is worth mentioning that some authors have reported more frequent medium changes [35, 45]. In summary, we present our observations on the effects of different protocol aspects, such as cell seeding density, coating, medium changes, or PBMC freezing, on the culture of MDMi cells. A more systematic study analyzing the functional properties of these cells would be important to corroborate these changes in the protocol.

### Microglial Nature of MDMi Cells. Morphology and Markers

One of the most noticeable features of MDMi cells reported in the original report by Ohgidani was their morphology. Phase contrast images showed cells with a small cell soma and many highly branched processes, strongly resembling the morphology of homeostatic microglial cells in the CNS parenchyma. This morphology, which is difficult to obtain in cultured microglia, has been observed when rodent microglia is co-cultured with astrocytes [49, 50], but very rarely in microglia cultured in isolation [51]. In this study, we have often observed this remarkable morphology, but cells with a less ramified morphology were also common. A close examination of the literature suggests that this occurs in most of the previous reports. Whereas many authors include a high magnification phase-contrast image showing microglial cells with a highly ramified morphology, careful examination of other figures shows that less ramified morphologies often predominate [32, 35, 44, 47]. To date, the most consistent ramified microglial morphology using this protocol is found in the publication by Quek et al. [46]. This highly ramified morphology is a defining feature of homeostatic microglia in the CNS parenchyma. However, it is important to note that it remains to be demonstrated that in vitro this morphology is associated with a more microglial phenotype than cells with less ramified morphologies.

Comparison of the expression of a series of microglial markers between MDMi and monocytes revealed that the differentiation protocol resulted in the upregulation of the microglial markers Iba1, P2RY12, and TMEM119 at the protein level and PROS1, GPR34, and P2RY12 at the mRNA level [52]. Such an increase was not observed for all microglial markers analyzed indicating that these cells cannot be considered *bona fide* microglial cells although they indeed have a microglia-like phenotype. The microglia-like phenotype of MDMi cells is strongly supported by reports that have compared the transcriptomic profile of MDMi cells with various microglia-related cells and have observed that MDMi cells separate from monocytes and human microglial cell lines and resemble resident human microglia [32, 35, 53].

### Functional Characterization: Phagocytosis and Proinflammatory Responses to LPS

A high phagocytic capacity is another defining feature of microglial cells. Here, we have demonstrated that MDMi cells can internalize α-synuclein fibrils in a conformation- and concentration-dependent manner. The 14 kDa protein α-synuclein is the main fibrillary component of Lewy bodies, one of the pathological hallmarks of Parkinson’s disease [54]. Its involvement in pathogenesis is further supported by the observation that point mutations as well as duplications and triplications of the α-synuclein gene SNCA are related to accelerated disease onset and progression in Parkinson’s disease [55]. The study of the internalization of α-synuclein by human microglia is relevant to understanding processes such as α-synuclein clearance [56], α-synuclein cell-to-cell transmission [57], and α-synuclein-induced inflammasome activation [58]. The results here obtained show that MDMi cells are a good model to study α-synuclein internalization by human microglia-like cells and are in line with previous studies showing that these cells also internalize fibrillar Aβ [35], bacteria [46], synaptosomes [32], or latex beads [47].

We have observed that the toll-like receptor 4 agonist LPS induces the rapid and transient nuclear translocation of the p65 subunit of the transcription factor NF-kappaB. The LPS-induced activation of NFkappaB is not a microglia-specific response [59], but it is a fundamental step in the activation of microglia by LPS and other proinflammatory stimuli [60] since NFkappaB is a master regulator of proinflammatory gene expression [61]. This response holds such functional significance that it should be reproduced in any experimental procedure aiming to model the so-called M1 activation of microglia, although this is not always the case [62]. Through the recruitment of NFkappaB and other transcription factors, LPS promotes the expression of proinflammatory genes in microglia. We have here studied a panel of 8 genes involved in this response. Most of them (TNF, IL6, IL1B, IL23A, CSF3, and PTGS2) were markedly upregulated at the mRNA level 6 h after LPS treatment and this increase was rapidly blunted, with mRNA levels almost returning to basal by 24 h. This is the prototypical response to LPS in this class of genes at the mRNA level and reflects the highly accessible chromatin structure in many proinflammatory genes that allows a rapid transcription initiation [63] and their characteristically short-lived mRNAs [64, 65]. As expected, LPS did not induce the upregulation of NOS2 mRNA levels in MDMi cells, since NOS2 is highly expressed by activated rodent microglia [41, 66] but not by human microglial cells [16, 17]. The activation of the proinflammatory gene program on microglial cells has been often associated with pathogenic consequences in many neurological disorders [67]. Our results therefore suggest that MDMi cells are a suitable model to study these responses in human microglia–like cells.

### Effect of C/EBPβ Inhibition on Proinflammatory Gene Expression in MDMi Cells

C/EBPβ is a transcription factor of the b-zip family that regulates proinflammatory gene expression in various cell types, including mouse microglia [68]. However, whether this transcription factor plays this role in human microglia has not yet been determined. Using a single administration of a pool of 4 siRNAs targeting human C/EBPΒ mRNA, we could induce a strong decrease in C/EBPΒ mRNA levels that was followed by a long-lasting (up to 14 days) decrease in C/EBPβ protein levels. These findings show that MDMi cells are amenable to specific gene inhibition by siRNAs. The decrease in C/EBPβ levels resulted in a marked attenuation of LPS-induced increased expression of important proinflammatory genes such as IL6, TNF, CSF3, CYBB, or PTGES. C/EBPβ regulates proinflammatory gene expression in murine microglial cells [7]. To our knowledge, these results show for the first time that C/EBPβ plays also this role in microglia-like cells of human origin and support the hypothesis that microglial C/EBPβ is a potential target to inhibit the detrimental effects of an exacerbated inflammatory response by microglia [5].

In summary, we have studied some properties of MDMi cells and have contributed to the characterization of the cell culture protocol. We found that a higher cell seeding density is preferable for establishing successful MDMi cell cultures; plate coating is not necessary, while GM-CSF and IL-34 are essential. Our results also show that MDMi cells are a good model to study proinflammatory responses and phagocytosis that are typical functional readouts used in studying microglia. MDMis were also suitable for silencing studies with siRNAs. Although MDMi cells express several microglial markers, these cells cannot be considered bona fide microglial cells since they show low expression of some microglial markers. The differences between MDMis and microglia may result in important nuances in their biology that could be unnoticed by the readouts used in this paper. Further studies are required to compare MDMis, stem cell–derived microglia-like cells, and primary human microglia to conclude whether there are important differences between bona fide microglia and these cellular models. In conclusion, MDMI cells are an interesting cellular model to study typical functional readouts used for microglial responses with many potential applications. Due to their accessibility and ease of cultivation, MDMi cells are particularly suitable for studying changes associated with human disease or human genetic variants.

## Supplementary Information

Below is the link to the electronic supplementary material.

Supplementary file1 (XLSX 69 KB)

Supplementary file2 (DOCX 2470 KB)

Supplementary file3 (DOCX 301 KB)

## Data Availability

The data that support the findings of this study are available from the corresponding author upon reasonable request.
