# Supplementary material for: Human Microglia–Like Cells Differentiated from Monocytes with GM-CSF and IL-34 Show Phagocytosis of α-Synuclein Aggregates and C/EBPβ-Dependent Proinflammatory Activation
Source: Mol Neurobiol. 2024 Jun 20;62(1):756–72. doi: 10.1007/s12035-024-04289-z (PMC11711251; doi:10.1007/s12035-024-04289-z)

**PROTOCOL FOR CULTURES OF MICROGLIA-LIKE FROM ADULT HUMAN BLOOD MONOCYTES**

By Andrea Llaves-López, Elia Micoli, Carla Belmonte-Mateos, Gerard Aguilar, Clara Alba, Anais Marsal, Marta Pulido-Salgado, Neus Rabaneda-Lombarte, Carme Solà, Joan Serratosa, Jose M Vidal-Taboada, Josep Saura. (School of Medicina, University of Barcelona, IIBB-CSIC, IDIBAPS)

*Adaptation of the protocol published in Ohgidani, M et al. Scientific Reports, 2014. See also Riedhammer et al (2015) Meth Mol Biol for isolation of PBMCs.*

**SOLUTIONS**

**RPMI -1640 medium:**

• 10% FBS (Invitrogen, 10270-106)

• Antibiotic and antimycotic: the article uses Antibiotic/antimycotic (100X) (Invitrogen, 15240-062 100ml) and corresponds to 100U/ml Penicillin, 100ug/ml Streptomycin and 0.25ug/ml Fungizone. To work under the same conditions, for 100 ml of medium we add:

• 1 ml of Penicillin (10,000 U/ml) - Streptomycin (10,000ug/ml) (aliquots -20ºCr) (Invitrogen, 15140-122)

• 0.1 ml of Fungizone 250ug/ml (aliquots -20ºC) (Invitrogen, 15290-018)

**Cytokines:**

• Human GM-CSF (R&D Systems, 215-GM-010). Stock 100ug/ml: resuspend the 10ug lyophilisate in 100ul of sterile PBS + 0.1% BSA. (It is aliquoted and frozen at -80°C (stable for 3 months). To work at 10ng/ml, add 1ul of the stock (100ug/ml) to 10 ml of medium.

• Human IL-34 (R&D Systems, 5265-IL-010). Stock 100ug/ml: resuspend the 10ug lyophilisate in 100ul of sterile PBS + 0.1% BSA. (It is aliquoted and frozen at -80°C (stable for 3 months). To work at 100ng/ml, add 10ul of the stock (100ug/ml) to 10ml of medium.

**BEFORE STARTING. CHECKLIST:**

□ Set the centrifuge to 18-20⁰C (turn it on and leave it at 18-20⁰C, it takes time to stabilize)

□ Have the Histopaque at RT (remove from the fridge about 30-45 minutes before starting)

□ Have the complete medium in the bath at 37°C (it can be prepared during step 3, but the RPMI bottle and FBS should be in the bath)

□ Have sterile H2O with Fz (Fungizone)

□ Check that we have the flasks, plates, hemocytometer, pipettes...

□ Double autoclave bag (cultures, cabinet under the sink). Everything that touches blood will be put in this bag, then autoclaved.

**PROTOCOL**

1. Three 50 mL vials are prepared, each with 20 mL of Histopaque-1077 (10771, Sigma-Aldrich), which must be at RT.

2. 60 mL of blood are extracted into tubes with EDTA (6 tubes of 10 mL with 18 mg EDTA) (367525, BD). Keep mixing them by inversion to prevent blood from coagulating.

*Each tube contains 9-9.5 ml of blood. According to Sigma, with Histopaque it is allowed: 15-30 U of heparin/ml blood and 1.25-1.75 mg EDTA/ml blood.*

3. A gradient is performed with Histopaque-1077 to separate the monocytes (10771, Sigma-Aldrich) (in the cold room). To each of the 3 falcons that have the Histopaque, add 20 mL of non-coagulated blood, very slowly, trying to mix as little as possible (very important!!). We do this by tilting a little the falcon where the Histopaque is, and with the pipette perpendicular. Centrifuge at 400g for 30 min at 18-20°C (aisle centrifuge, ~1400 rpm, brake off (this step is very important)).

*According to Riedhammer, centrifuging at temperatures >20°C gives worse performance because some lymphocytes (and monocytes?) aggregate with erythrocytes. Centrifugation at temperatures <18°C increases the density of the gradient medium and erythrocytes and granulocytes may appear in the PBMCs layer.*

4. During these 30 minutes, we prepare the medium (See "solutions" section). We need 50 mL of RPMI0 and 60 mL of RPMI10:

** 50 mL RPMI0 = 50 mL RPMI + 500 uL P/S + 50 uL Fz*

** 60 mL RPMI10, prepared in two separate falcons with 30mL each(27 mL RPMI + 3 mL FBS + 300 uL P/S + 30 uL Fz)*

When they are ready, we put them in the bathroom so that they are at 37°C when needed.


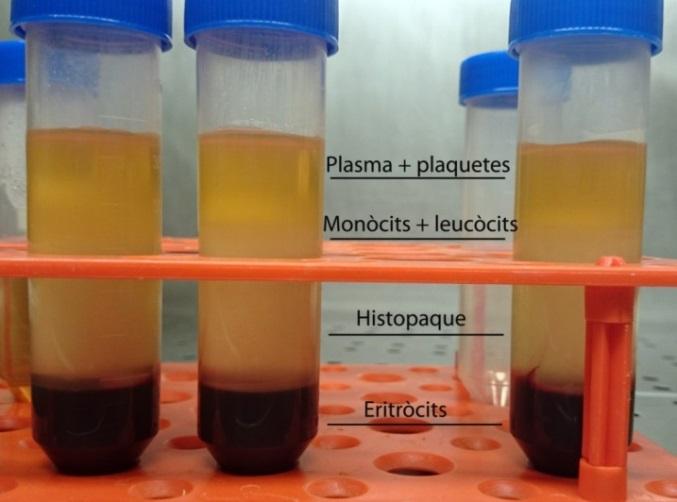
5. After centrifugation, the falcon tubes look like this:

We aspirate the layer of monocytes and leukocytes being careful not to take any solution from the other layers. From each Falcon tube, we recover 3 mL (in three aspirations of 1 mL with the p1000). Be careful not to disrupt the gradient while aspirating, because then in the second and third aspiration it is more difficult not to take material from the other layers. We place each interphase (every 3 mL of a falcon) in a 15 mL tube and add RPMI10 up to 14 mL. Mix by inversion about 3 times and centrifuge for 10 min at 200g (1200 rpm) at RT (culture centrifuge).

6. Remove the supernatant and put 1 mL of RPMI10 in each tube. We homogenize the pellet with the p100 (if a pellet is very difficult to disintegrate, we do not insist, we discard it, we think it is a platelet aggregate). We place the three resuspended pellets in one 15 mL tube and add medium up to 10 mL. Centrifuge for 10 min at 200g at RT.

7. Remove as much supernatant as possible without touching the pellet and resuspend the pellet in 1 mL of RPMI10. Add medium up to 5 mL and centrifuge for 10 min at 200g at RT.

8. Remove as much supernatant as possible without touching the pellet and resuspend the pellet in 5 mL of RPMI0.

9. Count the cells (only the bright ones; we obtained an average of 50-60x10^6^ cells/60 mL blood) and seed at a density of 1.6x10^6^ cells/ml. If we seed 48wp plates, seed only the central wells and add 300µl of H2O+Fungizone in the peripheral wells). Seeding volume in 48wp plates: 200 µl/well.

** The original protocol seeds at 0.4x10^5^ cells/ml; we have done tests at this density (x1), and at x2 and x4; cultures at x4 density were much better).*

** According to Riedhammer the typical yield is 0.5-2.5 x 10^6^ PBMCs per mL of blood. In our hands, this would be 0.8-1.6x10^6^ PBMCs per mL, that is, within the range.*

10. Keep the cells in the incubator overnight (37°C, 5% CO2). The next day, the medium is fully changed with 200µl/well of 48wp of RPMI0 supplemented with cytokines (see solutions section).

** Human GM-CSF 10ng/ml*

** Human IL-34 100ng/ml*

*It is quite inevitable that in addition to monocytes we have some platelets and non-adherent leukocytes. To remove them it is necessary to change the medium: stir the plate just before aspirating so that these cells rise and we take them when aspirating the medium.*

11. Cultivate the cells in standard conditions (37°C, 5% CO2) until DIV12-15 (considering DIV1 the day of seeding) with a full medium change at DIV7.

*In the first days cells are round and small. Over the days cells become very large. Cells can live without medium changes or additions for up to almost a month (this is explained in more detail in Andrea’s Master thesis). The next figure shows how the cells look at different times post-seeding (it is better to zoom).*


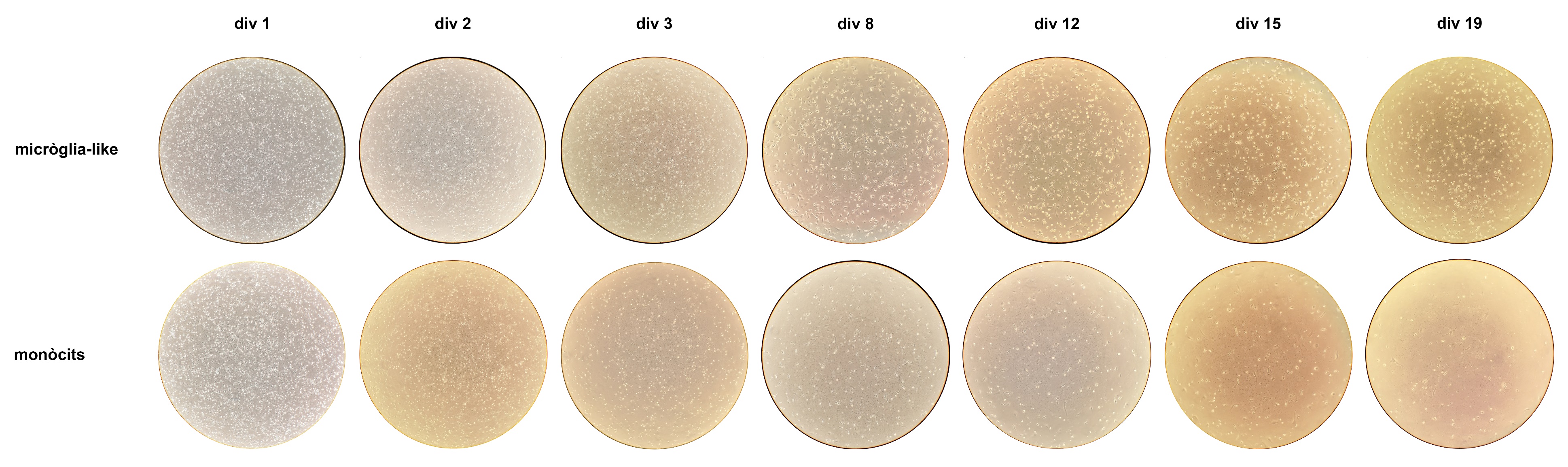


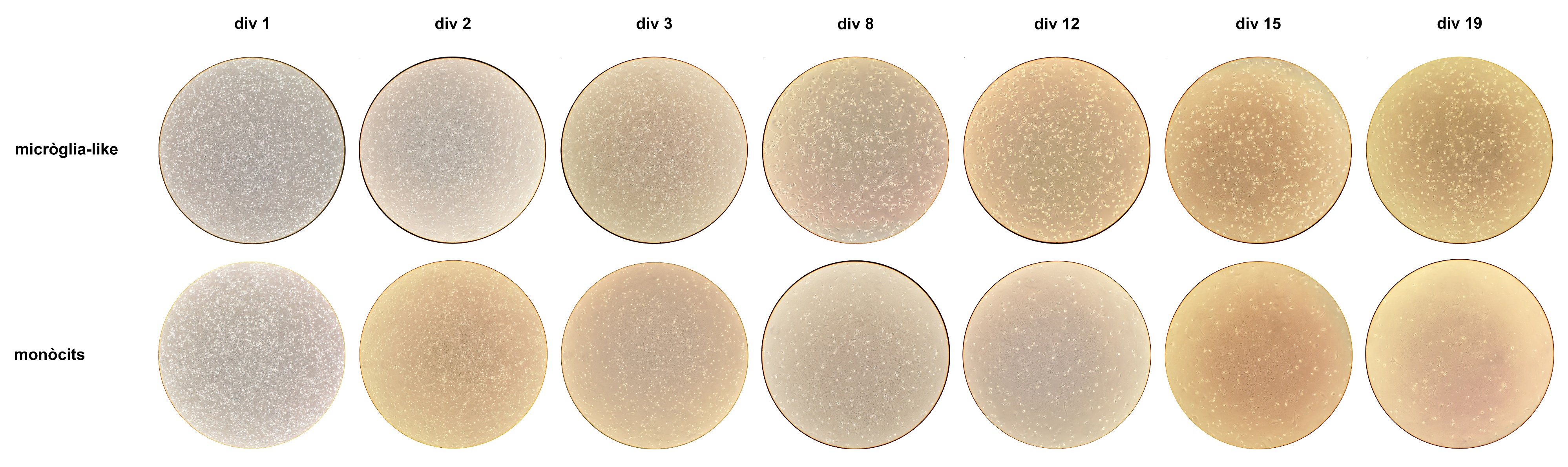

Supplement: Supplementary file 2 — Supplementary file2 (DOCX 2470 KB) [file 12035_2024_4289_MOESM2_ESM.docx]
