# Supplementary material for: Human Microglia–Like Cells Differentiated from Monocytes with GM-CSF and IL-34 Show Phagocytosis of α-Synuclein Aggregates and C/EBPβ-Dependent Proinflammatory Activation
Source: Mol Neurobiol. 2024 Jun 20;62(1):756–72. doi: 10.1007/s12035-024-04289-z (PMC11711251; doi:10.1007/s12035-024-04289-z)
Supplement: Supplementary file 3 — Supplementary file3 (DOCX 301 KB) [file 12035_2024_4289_MOESM3_ESM.docx]

Supplementary file 2. Amyloid fibril formation in aged α-synuclein samples was confirmed by transmission electron microscopy (TEM). Aged α-synuclein was prepared as indicated in the Methods section. Five μL from the aged α-synuclein solution were applied to carbon-coated copper grids, negatively stained with 1% (w/v) uranyl acetate, and visualized by TEM at the Electron Microscopy Facility Core of the Faculty of Medicine, UB.
